# Acceptance of a systematic review as a thesis: survey of biomedical doctoral programs in Europe

**DOI:** 10.1186/s13643-017-0653-x

**Published:** 2017-12-12

**Authors:** Livia Puljak, Damir Sapunar

**Affiliations:** 1 Cochrane Croatia, University of Split School of Medicine, Šoltanska 2, 21000 Split, Croatia; 2 Department for Development, Research and Health Technology Assessment, Agency for Quality and Accreditation in Health Care and Social Welfare, Planinska 13, 10000 Zagreb, Croatia; 3 Laboratory for Pain Research, University of Split School of Medicine, Šoltanska 2, 21000 Split, Croatia

**Keywords:** Systematic review, PhD program, Biomedicine, Study design, PhD thesis

## Abstract

**Background:**

Systematic reviews (SRs) have been proposed as a type of research methodology that should be acceptable for a graduate research thesis. The aim of this study was to analyse whether PhD theses in European biomedical graduate programs can be partly or entirely based on SRs.

**Methods:**

In 2016, we surveyed individuals in charge of European PhD programs from 105 institutions. The survey asked about acceptance of SRs as the partial or entire basis for a PhD thesis, their attitude towards such a model for PhD theses, and their knowledge about SR methodology.

**Results:**

We received responses from 86 individuals running PhD programs in 68 institutions (institutional response rate of 65%). In 47% of the programs, SRs were an acceptable study design for a PhD thesis. However, only 20% of participants expressed a personal opinion that SRs meet the criteria for a PhD thesis. The most common reasons for not accepting SRs as the basis for PhD theses were that SRs are ‘not a result of a PhD candidate’s independent work, but more of a team effort’ and that SRs ‘do not produce enough new knowledge for a dissertation’. The majority of participants were not familiar with basic concepts related to SRs; questions about meta-analyses and the type of plots frequently used in SRs were correctly answered by only one third of the participants.

**Conclusions:**

Raising awareness about the importance of SRs and their methodology could contribute to higher acceptance of SRs as a type of research that forms the basis of a PhD thesis.

**Electronic supplementary material:**

The online version of this article (10.1186/s13643-017-0653-x) contains supplementary material, which is available to authorized users.

## Background

Systematic reviews (SRs) are a type of secondary research, which refers to the analysis of data that have already been collected through primary research [1]. Even though SRs are a secondary type of research, a SR needs to start with a clearly defined research question and must follow rigorous research methodology, including definition of the study design a priori, data collection, appraisal of study quality, numerical analyses in the form of meta-analyses and other analyses when relevant and formulation of results and conclusions. Aveyard and Sharp defined SRs as ‘original empirical research’ because they ‘review, evaluate and synthesise all the available primary data, which can be either quantitative or qualitative’ [2]. Therefore, a SR represents a new research contribution to society and is considered the highest level in the hierarchy of evidence in medicine [3].

SRs have been proposed as a type of research methodology that should be acceptable as the basis for a graduate research thesis [4, 5]. To the best of our knowledge, there are no reports on the acceptance of SRs as the basis for PhD theses. A recent review addressed potential advantages and disadvantages of such a thesis type and presented opposing arguments about the issue [5]. However, there were no actual data that would indicate how prevalent one opinion is over another with regard to the acceptance of a SR as the primary research methodology for a PhD thesis. The aim of this cross-sectional study was to assess whether a PhD thesis in European biomedical graduate programs can be partly or entirely based on a SR, as well as to explore the attitudes and knowledge of individuals in charge of PhD programs with regard to a thesis of this type.

## Methods

### Participants

The Organization of PhD Education in Biomedicine and Health Sciences in the European System (ORPHEUS) includes 105 institutional members from 40 countries and six associate members from Canada, Georgia, Iran, Kyrgyzstan, Kazakhstan and the USA [6]. The ORPHEUS encompasses a network of higher education institutions committed to developing and disseminating best practice within PhD training programs in biomedicine, health sciences and public health. ORPHEUS approved the use of their mailing list for the purpose of this study. The mailing list had 1049 contacts. The study authors were not given the mailing list due to data protection and privacy. Instead, it was agreed that ORPHEUS officials would send the survey via email to the mailing list. The General Secretary of the ORPHEUS contacted individuals responsible for PhD programs (directors or deputy directors) among the institutional members, via e-mail, on 5th of July 2016. These individuals were sent an invitation to complete an online survey about SRs as the basis for PhD theses. We invited only individuals responsible for PhD programs (e.g., directors, deputy directors, head of graduate school, vice deans for graduate school or similar). We also asked them to communicate with other individuals in charge of their program to make sure that only one person per PhD program filled out the survey. If there were several PhD programs within one institution, we asked for participation of one senior person per program.

The survey was administered via Survey Monkey (Portland, OR, USA). The survey took 5–10 min to complete. One reminder was sent to the targeted participants 1 month after the first mail.

The ethics committee of the University of Split School of Medicine approved this study, which formed part of the Croatian Science Foundation grant no. IP-2014-09-7672 ‘Professionalism in Health Care’.

### Questionnaire

The 20-item questionnaire, designed specifically for this study by both authors (LP and DS), was first tested for face validity and clarity among five individuals in charge of PhD programs. The questionnaire was then modified according to their feedback. The questionnaire included questions about their PhD program; whether PhD candidates are required to publish manuscript(s) before thesis defence; the minimum number of required manuscripts for defending a PhD thesis; the authorship requirements for a PhD candidate with regard to published manuscript(s); whether there is a requirement for a PhD candidate to publish manuscript(s) in journals indexed in certain databases or journals of certain quality, and how the quality is defined; the description about other requirements for defending a PhD thesis; whether a SR partly or fully meets requirements for approval of a PhD thesis in their graduate program; what are the rules related to the use of a SR as the basis for a PhD thesis; and the number of PhD theses based on SRs relative to other types of research methods.

Participants were also asked about their opinion with regard to the main reasons that SRs are not recognised in some institutions as the basis for a doctoral dissertation, and their opinion about literature reviews, using a four-item Likert scale, ranging from ‘agree’ to ‘disagree’, including an option for ‘don’t know’. In the last question, the participants’ knowledge about SR methodology was examined using nine statements; participants had to rate each statement as either ‘correct’, ‘incorrect’, ‘unsure’ or ‘I don’t know’. Finally, participants were invited to leave their email address if they wanted to receive survey results. The survey sent to the study participants can be found in an additional file (Additional file 1).

### Data analysis

Survey responses were entered into a spreadsheet, checked by both authors and analysed using Microsoft Excel (Microsoft Inc., Redmond, WA, USA). Descriptive data are presented as frequencies and percentages. All raw data and analysed data sets used in the manuscript are available from authors on request. A point-biserial correlation (SPSS, IBM, Chicago, IL, USA) was used to measure the strength of the association between results on the knowledge test (continuous variable) and the attitude towards SRs as the basis for dissertations (dichotomous variable; we used the answer to the following question as this measure: ‘Do you agree that a systematic review, in whole or in part, meets the criteria for a publication on which a doctoral dissertation can be based?’).

## Results

### Study participants

There are 105 institutions included in the ORPHEUS network. We received a response from 86 individuals representing 68 institutions from 37 countries (65% institutional response rate). There were more respondents than institutions because some institutions have several PhD programs and thus several program directors. Those responders were used as a unit of analysis in the analysis of attitudes and knowledge; institutions were the unit of analysis when analysing criteria for theses. Some of the questionnaires (*n* = 15) were only partly completed. In most cases, the missing data were related to knowledge about SR methodology.

### Overview of requirements for a dissertation

Based on the information provided by the graduate program directors, in the majority of the included PhD programs, students were required to publish a research manuscript prepared within their PhD thesis prior to their thesis defence (83%; *n* = 64). Among 13 programs (17%) that did not have this requirement, five respondents (38%) indicated that in their opinion their school’s rules related to a PhD thesis should be changed such as to specify that each thesis should be based on work that is already published in a journal.

The minimum number of published manuscripts necessary for the PhD thesis defence was prespecified in 94% (*n* = 60) of the programs that required publication of research manuscripts prior to the thesis defence. In most of the programs (37%; *n* = 22), the number of required manuscripts was three or more. Two manuscripts were required in 30% (*n* = 18) and one was required in 33% (*n* = 20) of the programs. In four programs, there was no formal policy on this matter, but there was a strong expectation that the student will have contributed substantially to several manuscripts in peer-reviewed journals.

In most cases, the PhD candidates’ contribution to published manuscripts within the PhD thesis was determined through first authorship. A requirement that a PhD candidate should be the first author on a manuscript(s) that constitutes a PhD thesis was reported in 82% (*n* = 64) of the graduate programs.

In 60% (*n* = 52) of the graduate programs, the quality of the journals where a PhD candidate has to publish research manuscripts as a part of a PhD thesis was defined by the database in which these journals are indexed. The most commonly specified databases were Web of Science (41%; *n* = 35) and MEDLINE/PubMed (13%; *n* = 11), followed by Science Citation Index, Scopus, Current Contents, a combination of several databases or, in two cases, a combination of journals from a list defined by some governing body.

### Systematic reviews as a PhD thesis

**Fig. 1:**
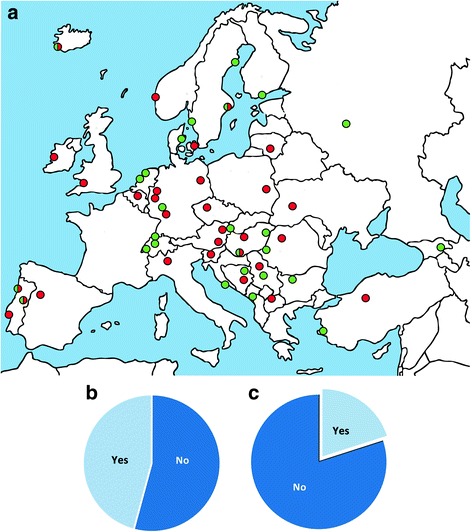
**a** European PhD programs that recognise a systematic review as a PhD thesis (green dot) and those that do not (red dot). Half red and half green dots indicate the five universities with institutions that have opposite rules regarding recognition of a systematic review as a PhD thesis. The pie chart presents **b** the percentage of the programs in which systematic reviews, in whole or in part, meet the criteria for a dissertation and **c** the opinion of participants about whether systematic reviews should form the basis of a publication within a PhD dissertation

SRs, in whole or in part, met the criteria for acceptable research methodology for a PhD thesis in 47% (*n* = 40) of programs, whereas 53% (*n* = 46) of programs specifically stated that they did not accept SRs in this context (Fig. 1a, b). Among the programs that accepted SRs, theses could be exclusively based on a SR in 42% (*n* = 17) of programs, while in the remaining programs, SRs were acceptable as one publication among others in a dissertation.

The majority of participants (80%; *n* = 69) indicated that SRs did not meet criteria for a publication on which a PhD dissertation should be based (Fig. 1c). The main arguments for not recognising a SR as the basis for a PhD thesis are listed in Table 1. The majority of respondents were neutral regarding the idea that scoping reviews or SRs should replace traditional narrative reviews preceding the results of clinical and basic studies in doctoral theses. Most of the respondents agreed that narrative or critical/discursive literature reviews preceding clinical studies planned as part of a dissertation should be replaced with systematic reviews (Table 2).

**Table 1:** The main reasons for not recognising a systematic review as the basis for a PhD thesis in European biomedical graduate programs

Survey items	Agree *n* (%)	Neither agree nor disagree *n* (%)	Disagree *n* (%)	Don’t know *n* (%)
Systematic reviews are not a result of the candidate’s independent work since systematic reviews tend to be conducted by a team	41 (57.7%)	11 (15.5%)	11 (15.5)	8 (11.3%)
Systematic reviews do not produce enough new knowledge for a dissertation	38 (53.5%)	8 (11.3%)	20 (28.2%)	5 (7.0%)
Because of a concern arising when there are no primary studies available on a particular topic, or the inclusion criteria are too narrow (‘empty reviews’)	22 (31.0%)	26 (36.6%)	11 (15.5%)	12 (16.9%)
Systematic reviews are too easy to perform	22 (31.0%)	14 (18.7%)	31 (43.6%)	4 (5.6%)
There are no major differences between classical narrative and systematic reviews	14 (18.7%)	12 (16.9%)	37 (2.1%)	8 (11.3%)
Lack of expertise among committee members regarding systematic reviews, since they should be experienced in systematic review methodology	24 (33.8%)	22 (31.0%)	18 (25.4%)	7 (9.9%)
Lack of adequate training of candidates in methodology of systematic reviews	33 (46.5%)	19 (26.8%)	15 (21.1%)	6 (8.5%)
Students are not experienced enough to perform critical analysis of primary studies	31 (43.7%)	17 (23.9%)	18 (25.4%)	5 (7.0%)
Lack of appreciation of systematic review methodology among faculty members	25 (35.0%)	23 (32.0%)	18 (25.0%)	5 (7.0%)

**Table 2:** Respondents’ opinions about literature reviews

Survey items	Agree *n* (%)	Neither agree nor disagree *n* (%)	Disagree *n* (%)	Don’t know *n* (%)
Narrative or critical/discursive literature reviews preceding clinical studies planned as part of a dissertation should be replaced with scoping reviews	16 (22.5%)	27 (38.0%)	19 (26.8%)	9 (12.7%)
Narrative or critical/discursive literature reviews preceding clinical studies planned as part of a dissertation should be replaced with systematic reviews	23 (32.4%)	24 (33.8%)	18 (25.4%)	6 (8.5%)
Narrative or critical/discursive literature reviews preceding basic studies planned as part of a dissertation should be replaced with scoping reviews	17 (23.9%)	26 (36.6%)	21 (29.6%)	7 (9.9%)
Narrative or critical/discursive literature reviews preceding basic studies planned as part of a dissertation should be replaced with systematic reviews	18 (25.4%)	25 (35.2%)	23 (32.4%)	5 (7.0%)

**Fig. 2:**
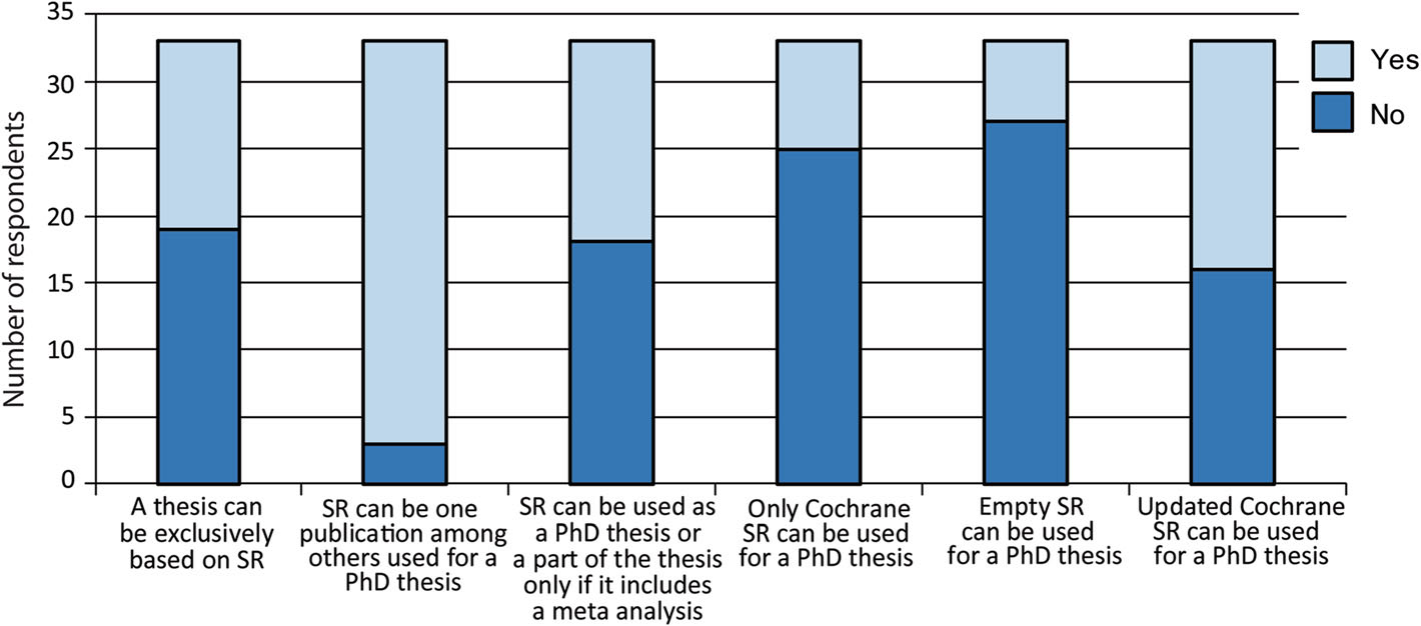
Frequency of different rules that define the use of systematic reviews as a part of a PhD thesis in European biomedical graduate programs

Most of the programs that accepted SRs as a research methodology acceptable for PhD theses had defined rules related to the use of an SR as part of a PhD thesis (Fig. 2). The most common rule was that a SR can be one publication among others within a PhD thesis. Some of the respondents indicated that empty (reviews that did not find a single study that should be included after literature search) or updated reviews could also be used for a PhD thesis (Fig. 2).

The results of the survey regarding knowledge about SR methodology indicated that the majority of respondents were not familiar with this methodology. Only three out of nine questions were correctly answered by more than 80% of the participants, and questions about meta-analyses and the type of plots frequently used in a SR were correctly answered by only one third of the participants (Table 3). The association between participants’ results on the knowledge test and attitudes towards SRs was tested using a point-biserial correlation; this revealed that lack of knowledge was not correlated with negative attitudes towards SRs (*r*_pb_ = 0.011; *P* = 0.94).

**Table 3:** Knowledge of systematic reviews among individuals in charge of European biomedical graduate programs

Survey items	Yes *n* (%)	No *n* (%)	I’m not sure *n* (%)	I don’t know *n* (%)
It is sufficient to search one database to produce a systematic review (correct answer: no)	6 (8.6%)	57 (80.3%)	5 (7.0%)	3 (4.2%)
Systematic reviews must be produced by one author only (correct answer: no)	4 (5.6%)	57 (80.3%)	6 (8.5%)	4 (5.6%)
Systematic reviews must contain meta-analyses (correct answer: no)	31 (43.7%)	22 (31.0%)	15 (21.1%)	3 (4.2%)
Systematic reviews must have duplicate screening and data extraction (correct answer: yes)	30 (42.3%)	3 (4.2%)	25 (35.2%)	13 (18.3%)
A list of both included and excluded studies must be provided (correct answer: yes)	48 (67.6%)	5 (7.0%)	13 (18.3%)	5 (7.0%)
Quality of included studies must be assessed (correct answer: yes)	60 (84.5%)	4 (5.6%)	4 (5.6%)	3 (4.2%)
In the case of meta-analyses, a heterogeneity test must be done to ensure the results of studies can be combined (correct answer: yes)	46 (64.8%)	3 (4.2%)	13 (18.3%)	9 (12.7%)
Results of meta-analyses must be presented as a funnel plot (correct answer: no)	26 (36.6%)	7 (9.9%)	25 (35.2%)	13 (18.3%)
Results of publication bias analysis must be presented as a forest plot (correct answer: no)	23 (32.4%)	8 (11.3%)	27 (38.0%)	13 (18.3%)

## Discussion

In this study conducted among individuals in charge of biomedical graduate programs in Europe, we found that 47% of programs accepted SRs as research methodology that can partly or fully fulfil the criteria for a PhD thesis. However, most of the participants had negative attitudes about such a model for a PhD thesis, and most had insufficient knowledge about the basic aspects of SR methodology. These negative attitudes and lack of knowledge likely contribute to low acceptance of SRs as an acceptable study design to include in a PhD thesis.

A limitation of this study was that we relied on participants’ responses and not on assessments of formal rules of PhD programs. Due to a lack of familiarity with SRs, it is possible that the respondents gave incorrect answers. We believe that this might be the case since we received answers from different programs in the same university, where one person claimed that SRs were accepted in their program, and the other person claimed that they were not accepted in the other program. We had five such cases, so it is possible that institutions within the same university have different rules related to accepted research methodology in graduate PhD programs. This study may not be generalisable to different PhD programs worldwide that were not surveyed. The study is also not generalisable to Europe, as there are no universal criteria or expectations for PhD theses in Europe. Even in the same country, there may be different models and expectations for a PhD in different higher education institutions.

A recent study indicated a number of opposing views and disadvantages related to SRs as research methodology for graduate theses, including lack of knowledge and understanding by potential supervisors, which may prevent them from being mentors and assisting students to complete such a study [5]. This same manuscript emphasised that there may be constraints if the study is conducted in a resource-limited environment without access to electronic databases, that there may be a very high or very low number of relevant studies that can impact the review process, that methods may not be well developed for certain types of research syntheses and that it may be difficult to publish SRs [5].

Some individuals believe that a SR is not original research. Indeed, it has been suggested that SRs as ‘secondary research’ are different than ‘primary or original research’, implying that they are inferior and lacking in novelty and methodological rigour as compared to studies that are considered primary research. In 1995, Feinstein suggested that such studies are ‘statistical alchemy for the 21st century’ and that a meta-analysis removes or destructs ‘scientific requirements that have been so carefully developed and established during the 19th and 20th centuries’ [7]. There is little research about this methodological issue. Meerpohl et al. surveyed journal editors and asked whether they consider SRs to be original studies. The majority of the editors indicated that they do think that SRs are original scientific contributions (71%) and almost all journals (93%) published SRs. That study also highlighted that the definition of original research may be a grey area [8]. They argued that, in an ideal situation, ‘the research community would accept systematic reviews as a research category of its own, which is defined by methodological criteria, as is the case for other types of research’ [8]. Biondi-Zoccai et al. pointed out that the main criteria to judge a SR should be its novelty and usefulness, and not whether it is original/primary or secondary research [9].

In our study, 80% of the participants reported negative attitudes, and more than half of the respondents agreed with a statement that SRs are ‘not a result of the candidate’s independent work since systematic reviews tend to be conducted by a team’. This opinion is surprising since other types of research are also conducted within a team, and single authorship is very rare in publications that are published within a PhD thesis. On the contrary, the mean number of authors of research manuscripts is continuously increasing [10]. At the very least, the authors of manuscripts within a PhD will include the PhD candidate and a mentor, which is a team in and of itself. Therefore, it is unclear why somebody would consider it a problem that a SR is conducted within a team.

The second most commonly chosen argument against such a thesis was that SRs ‘do not produce enough new knowledge for a dissertation’. The volume of a SR largely depends on the number of included studies and the available data for numerical analyses. Therefore, it is unfair to label a SR as a priori lacking in new knowledge. There are SRs with tens or hundreds of included studies, and some of them not only include meta-analyses, but also network meta-analyses, which are highly sophisticated statistical methods. However, limiting SRs within a thesis only to those with meta-analysis would be unfair because sometimes meta-analysis is not justified due to clinical or statistical heterogeneity [11] and the presence or absence of a meta-analysis is not an indicator of the quality of a SR. Instead, there are relevant checklists for appraising methodological and reporting quality of a SR [12, 13].

The third most commonly chosen argument against SRs within PhD theses was ‘lack of adequate training of candidates in methodology of systematic reviews’. This could refer to either insufficient formal training or insufficient mentoring. The graduate program and the mentor need to ensure that a PhD candidate receives sufficient knowledge to complete the proposed thesis topic. Successful mentoring in academic medicine requires not only commitment and interpersonal skills from both the mentor and mentee, but also a facilitating institutional environment [14]. This finding could be a result of a lack of capacity and knowledge for conducting SRs in the particular institutions where the survey was conducted, and not general opinion related to learning a research method when conducting a PhD study. Formal training in skills related to SRs and research synthesis methods [15, 16], as well as establishing research collaborations with researchers experienced in this methodology, could alleviate this concern.

One third of the participants indicated a ‘lack of appreciation of systematic review methodology among faculty members’ as a reason against such a thesis model. This argument, as well as the prevalent negative attitude towards SRs as PhD theses, perhaps can be traced to a lack of knowledge about SR methodology; however, although the level of knowledge was quite low in our study, there was no statistically significant correlation between knowledge and negative attitudes. Of the nine questions about SR research methodology, only three questions were correctly answered by more than half of the participants. This could be a cause for concern because it has been argued that any health research should begin with a SR of the literature [17]. It has also been argued that the absence of SRs in the context of research training might severely hamper research trainees and may negatively impact the research conducted [18]. Thus, it has been recommended that SRs should be included ‘whenever appropriate, as a mandatory part of any PhD program or candidature’ [18].

It has recently been suggested that the overwhelming majority of investment in research represents an ‘avoidable waste’ [19]. Research that is not necessary harms both the public and patients, because funds are not invested where they are really necessary, and necessary research may not be conducted [17]. This is valid not only for clinical trials, but also for other types of animal and human experiments [20]. SRs can help improve the design of new experiments by relying on current evidence in the field and by helping to clarify which questions still need to be addressed. SRs can be instrumental in improving methodological quality of new experiments, providing evidence-based recommendations for research models, reducing avoidable waste, and enabling evidence-based translational research [20].

Four respondents from three institutions indicated that empty SRs are accepted as a PhD thesis. While it makes sense to include such a SR as a part of the thesis to indicate lack of evidence in a certain field, it is highly unlikely that an entire thesis can be based on an empty SR, without a single included study.

There are many advantages of a SR as a graduate thesis [4, 5], especially as a research methodology suitable for low-resource settings. A PhD candidate can prepare a Cochrane SR as a part of the PhD thesis, yielding a high-impact publication [4]. Non-Cochrane SRs can also be published in high-impact journals. A PhD candidate involved in producing a SR within a PhD thesis goes through the same research process as those conducting primary research, from setting up a hypothesis and a research question, to development of a protocol, data collection, data analysis and appraisal, and formulation of conclusions. Graduate programs can set limits, such as the prevention of empty reviews and the recognition of updated reviews as valid for a PhD thesis, and engage experienced researchers as advisors and within thesis evaluation committees, to ensure that a candidate will conduct a high-quality SR [4]. Conducting a SR should not be mandatory, but candidates and mentors willing to produce such research within a graduate program should be allowed to do so.

Further studies in this field could provide better insight into attitudes related to SRs as graduate theses and explore interventions that can be used to change negative attitudes and improve knowledge of SRs among decision-makers in graduate education.

## Conclusions

Raising awareness about the importance of SRs in biomedicine, the basic aspects of SR methodology and the status of SRs as original secondary research could contribute to greater acceptance of SRs as potential PhD theses. Our results can be used to create strategies that will enhance acceptance of SRs among graduate education program directors.

## Additional file

Additional file 1: Online survey used in the study. Full online survey that was sent to the study participants. (PDF 293 kb)
