# Supplementary material for: Acceptance of a systematic review as a thesis: survey of biomedical doctoral programs in Europe
Source: Syst Rev. 2017 Dec 12;6:253. doi: 10.1186/s13643-017-0653-x (PMC5727923; doi:10.1186/s13643-017-0653-x)
Supplement: Additional file 1: — Online survey used in the study. Full online survey that was sent to the study participants. (PDF 293 kb) [file 13643_2017_653_MOESM1_ESM.pdf]

Orpheus Survey: Systematic Review as a PhD thesis

Dear ORPHEUS members,
Below is a short survey to collect information on systematic reviews in the context of doctoral education and doctoral theses. The survey has been designed as a part of a research project "Professionalism in Health Care", funded by the Croatian Research Foundation. The findings from the survey will be used to improve our programs and will be presented during the next ORPHEUS conference.
Your willingness to cooperate is highly appreciated. Your input will be treated with full confidentiality and anonymity, as your personal data will not be collected. By continuing to the survey questions you consent to participate in it.
You can include your contact details on the last page if you are interested in receiving a copy of the survey results.
Kind regards,
Damir Sapunar
Livia Puljak
Directors of the TRIBE PhD program
University of Split, School of Medicine, Split, Croatia
www.mefst.hr/TRIBE

* 1. What is the name of your PhD / doctoral program?

* 2. What is the name of your university?

* 3. What is the name of your school / faculty?

* 4. What is your role in the PhD / doctoral program?

* 5. Are PhD candidates enrolled in your PhD / doctoral program required to publish a research article prepared within their doctoral theses prior to the thesis defense?

- ○ Yes
- ○ No

TRIBE
TRANSLATIONAL RESEARCH IN BIOMEDICINE

Orpheus Survey: Systematic Review as a PhD thesis

* 6. Is there a minimum number of published articles required for a dissertation?

- ○ Yes
- ○ No

7. If you answered Yes to the previous question, what is the number of published articles required?

[ ]

* 8. Is there a requirement for the first authorship on research articles that constitute a dissertation?

- ○ Yes
- ○ No

9. If you answered Yes to the previous question, what is the number of first authored articles necessary for a dissertation?

[ ]

* 10. Is there a requirement to publish in journals listed in certain databases (WoS, Current Contents, Scopus...)?

- ○ Yes
- ○ No

11. If you answered Yes to the previous question, please list all databases that apply.

[ ]

* 12. Is there a requirement for publishing in journals of a certain quality?

◯ Yes

◯ No

13. If you answered Yes to the previous question, how is the quality of journal defined (quartiles, IF…) and what is the acceptable level of quality?

[ ]

14. Please describe in brief if there are any other requirements for the thesis defense related to research articles.

[ ]

* 15. Does a published systematic review, in whole or in part, meet the criteria for a dissertation at your school?

◯ Yes

◯ No

TRIBE
TRANSLATIONAL RESEARCH IN BIOMEDICINE

Orpheus Survey: Systematic Review as a PhD thesis

* 16. What are the rules related to the use of systematic reviews as a part of a doctoral dissertation?

	Yes	No
A thesis can be exclusively based on systematic reviews	○	○
Systematic reviews can be one publication among others used for a dissertation	○	○
Systematic review can be used as a doctoral thesis or a part of the doctoral thesis only if it includes a meta analysis	○	○
Only Cochrane systematic reviews can be used for a dissertation	○	○
Empty systematic reviews can be used for a dissertation	○	○
Updated Cochrane systematic reviews can be used for a dissertation	○	○

* 17. The number of dissertations based on systematic reviews at your school / program is:

- ○ Lower than the number of dissertatins based on other types of studies
- ○ Equal to the number of dissertations based on other types of studies
- ○ Higher than the number of dissertatins based on other types of studies

TRIBE
TRANSLATIONAL
RESEARCH
IN BIOMEDICINE

Orpheus Survey: Systematic Review as a PhD thesis

* 18. In your opinion, should the bylaws at your school be changed to introduce the rule that each thesis should be based on work that was already publish in a journal.

- Yes
- No

Orpheus Survey: Systematic Review as a PhD thesis

* 19. Do you agree that a systematic review, in whole or in part, meets the criteria as a publication on which a doctoral dissertation can be based?

- Yes
- No

* 20. In your opinion, what are the main reasons why systematic reviews are not recognized in some institutions as a basis for the doctoral dissertation?

	Strongly agree	Agree	Neither agree or disagree	Disagree	Strongly disagree	Don't know
Systematic reviews are not a result of the candidate's independent work since systematic reviews tend to be conducted by a team	◯	◯	◯	◯	◯	◯
Systematic reviews do not produce enough new knowledge for a dissertation	◯	◯	◯	◯	◯	◯
Because of a concern arising when there are no primary studies available on a particular topic, or the inclusion criteria are too narrow (issues "empty reviews")	◯	◯	◯	◯	◯	◯
Systematic reviews are too easy to perform	◯	◯	◯	◯	◯	◯
There are no major differences between classical narrative and systematic reviews	◯	◯	◯	◯	◯	◯
Lack of expertise among committee members regarding systematic reviews, since they should be experienced in systematic review methodology	◯	◯	◯	◯	◯	◯
Lack of adequate training of candidates in methodology of systematic reviews	◯	◯	◯	◯	◯	◯
Students are not experienced enough to perform critical analysis of primary studies	◯	◯	◯	◯	◯	◯
Lack of appreciation of systematic review methodology among faculty members	◯	◯	◯	◯	◯	◯

* 21. What is your opinion on literature reviews?

	Strongly agree	Agree	Neither agree or disagree	Disagree	Strongly disagree	Don't know
Narrative, or critical/discursive literature reviews preceding **clinical studies** planned as a part of dissertation should be replaced with **scoping reviews**	○	○	○	○	○	○
Narrative, or critical/discursive literature reviews preceding **clinical studies** planned as a part of dissertation should be replaced with **systematic reviews**	○	○	○	○	○	○
Narrative, or critical/discursive literature reviews preceding **basic studies** planned as a part of dissertation should be replaced with **scoping reviews**	○	○	○	○	○	○
Narrative, or critical/discursive literature reviews preceding **basic studies** planned as a part of dissertation should be replaced with **systematic reviews**	○	○	○	○	○	○

* 22. What are the characteristics of a good systematic review?

	Yes	No	I am not sure	I don't know
It is sufficient to search one database to produce a systematic review	◯	◯	◯	◯
Systematic review must be produced by one author only	◯	◯	◯	◯
Systematic review must contain meta-analysis	◯	◯	◯	◯
Systematic review must have duplicate screening and data extraction	◯	◯	◯	◯
A list of both included and excluded studies must be provided	◯	◯	◯	◯
Quality of included studies must be assessed	◯	◯	◯	◯
In the case of meta-analysis, a heterogeneity test must be done to ensure the results of studies can be combined	◯	◯	◯	◯
Results of meta-analysis must be presented as a funnel plot	◯	◯	◯	◯
Results of publication bias analysis must be presented as a forest plot	◯	◯	◯	◯

23. Please include your mail address if you are interested in receiving a copy of the survey results.
